# Finding miRNA–RNA Network Biomarkers for Predicting Metastasis and Prognosis in Cancer

**DOI:** 10.3390/ijms24055052

**Published:** 2023-03-06

**Authors:** Seokwoo Lee, Myounghoon Cho, Byungkyu Park, Kyungsook Han

**Affiliations:** 1 Department of Computer Engineering, Inha University, Incheon 22212, Republic of Korea; 2 Research and Development Center, Hancom Carelink Incorporated, Seongnam 13493, Republic of Korea

**Keywords:** miRNA–RNA interaction, patient-specific network, differential correlation, cancer, prognosis, metastasis

## Abstract

Despite remarkable progress in cancer research and treatment over the past decades, cancer ranks as a leading cause of death worldwide. In particular, metastasis is the major cause of cancer deaths. After an extensive analysis of miRNAs and RNAs in tumor tissue samples, we derived miRNA–RNA pairs with substantially different correlations from those in normal tissue samples. Using the differential miRNA–RNA correlations, we constructed models for predicting metastasis. A comparison of our model to other models with the same data sets of solid cancer showed that our model is much better than the others in both lymph node metastasis and distant metastasis. The miRNA–RNA correlations were also used in finding prognostic network biomarkers in cancer patients. The results of our study showed that miRNA–RNA correlations and networks consisting of miRNA–RNA pairs were more powerful in predicting prognosis as well as metastasis. Our method and the biomarkers obtained using the method will be useful for predicting metastasis and prognosis, which in turn will help select treatment options for cancer patients and targets of anti-cancer drug discovery.

## 1. Introduction

The past two decades have seen remarkable progress in cancer research and treatment. However, despite significant progress, cancer still affects millions of people and ranks as a leading cause of death in the world [1]. In particular, metastasis is the major cause of cancer mortality, which accounts for about 90% of cancer deaths [2,3]. Cancer is a complex and heterogeneous disease with many possible genetic and environmental causes. Many treatments are effective only for patients with specific genetic or epigenetic alterations that help tumor cells develop [4,5]. Therefore, finding genetic changes specific to individual patients is essential to selecting effective treatments for cancer patients [6].

In our previous studies [7,8], we have developed a method for constructing microRNAs (miRNAs) mediated RNA interaction networks specific to individual cancer patients and for finding prognostic miRNA–RNA pairs or lncRNA–miRNA–mRNA triplets. A miRNA is a small non-coding RNA molecule of ~22 nucleotides, which often represses the expression of a gene by binding to the gene [9]. Until recently, interactions between miRNAs and their target genes have not received much attention from cancer research scientists. The so-called competitive endogenous RNA (ceRNA) hypothesis proposed by Salmena et al. [10] suggests that miRNAs mediate a regulatory relation between long non-coding RNAs (lncRNAs) and mRNAs which share similar miRNA response elements (MREs) to bind to the same miRNA. Results of several experimental studies have supported the hypothesis and demonstrated that many miRNAs are key regulators in the initiation and development of cancer [11,12,13,14,15]. The ceRNA hypothesis focused on competing relations between lncRNAs and mRNAs only, but competition for miRNA-binding occurs not only between lncRNAs and mRNAs but also between lncRNAs or between mRNAs. Furthermore, many pseudogenes also act as ceRNAs, thereby regulating other genes.

Motivated by the increasing amount of miRNA expression data, several studies have been conducted recently to construct ceRNA networks in cancer. Zhu et al. [16], for example, constructed a network of lncRNA–miRNA–mRNA triplets from miRNA–lncRNA associations and miRNA–mRNA associations. Jiang et al. [17] constructed a ceRNA network after calculating the correlation coefficients of miRNA-mRNA and miRNA–lncRNA pairs. However, most ceRNA networks constructed so far are intended to represent a general relation of RNAs present in multiple cancer samples rather than for a patient-specific relation of RNAs. The biological functions of the regulatory miRNAs are very diverse depending on the target molecules regulated by miRNAs. In particular, cancer is a very heterogeneous disease, so RNA interactions mediated by miRNAs can vary in different cancer patients.

As an extension of our previous studies [7,8], we have developed a new method of finding biomarkers based on differential miRNA–RNA correlations to predict metastasis and prognosis in cancer. Unlike conventional molecular biomarkers, network biomarkers can capture the associations or regulations of molecules involved in complex diseases such as cancer [18]. A network-based approach is one of the emerging promising strategies, and the transition from molecular biomarkers to network biomarkers will help select treatment options tailored to individual patients. The rest of this paper presents our approach to deriving miRNA–RNA correlations specific to cancer patients and finding biomarkers for predicting metastasis and prognosis.

## 2. Results and Discussion

### 2.1. miRNA–RNA Pairs

Table 1 shows the number of tumor samples of each type and normal samples in 10 cancer data sets. In most cancer data sets, there were many fewer tumor samples with distant metastasis than tumor samples with lymph node metastasis.

The RNAs of 4 biotypes (miRNAs, lncRNAs, mRNAs, and pseudogenes) obtained after removing those with low-expressions are shown in Table 2. The number of miRNA–RNA pairs left after each filtering process is shown in Table 3. The correlations of the miRNA–RNA pairs were used in our study to predict metastasis and prognosis.

### 2.2. Prediction of Metastasis and Comparison with Other Methods

While our method uses ΔPCCs of miRNA–RNA pairs as features, most learning-based methods for predicting metastasis use gene expressions as features. We compared the performance of prediction using three different types of features: ΔPCCs of miRNA–RNA pairs, expressions of genes involved in miRNA–RNA pairs, and expressions of 191 metastasis-predictive genes found by Zhou et al. [19].

For a fair comparison, three methods with different features were evaluated in the same way. We partitioned the data sets randomly into training and test data sets with a ratio of 7:3. We used the training data set to optimize the hyperparameters of each model using a grid search with 5-fold cross-validation. We repeated the whole process of the data partition, training, and testing 10 times for the evaluation of the methods.

**Figure 1:**
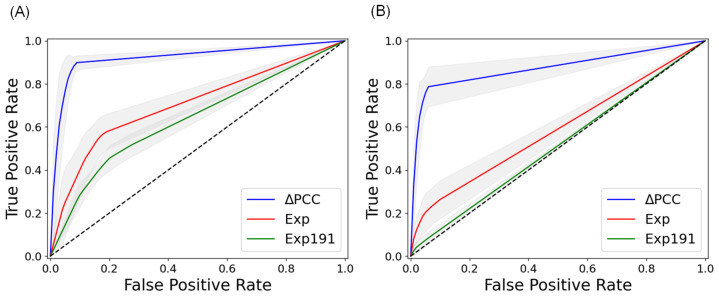
ROC curves of three types of features in predicting metastasis of COAD in independent testing. (**A**) Average ROC curves in predicting lymph node metastasis of COAD. (**B**) Average ROC curves in predicting distant metastasis of COAD. ΔPCC: ΔPCC of miRNA–RNA pairs, Exp: Expressions of genes involved in miRNA–RNA pairs, Exp191: Expressions of 191 metastasis-predictive genes [19]. The curves are the average ROC curves of 10 runs. The gray part indicates the error range of the ROC curves.

Table 4 shows the average area under the curve (AUC) values of the three methods with independent data sets, which were not used in training the methods. Our method, which used ΔPCCs of miRNA–RNA pairs, outperformed the other methods in all 10 cancer types. Figure 1 compares the average ROC curves of the three methods with independent data sets of COAD. It is interesting to note that the 191 metastasis-predictive genes were not predictive of prognosis in both distant metastasis and lymph node metastasis. The results demonstrate that ΔPCCs of miRNA–RNA interactions are more powerful than gene expressions in predicting lymph node metastasis and distant metastasis. Detailed results of 5-fold cross-validation and independent testing of the three methods are available in Appendix A.

### 2.3. Predicting Prognosis and Potential Prognostic Biomarkers

We performed the univariate Cox regression analysis with respect to |ΔPCC| values of miRNA–RNA pairs to explore the overall survival of patients. Table 5 shows the top miRNA–RNA pairs with the lowest *p*-value of the log–rank test in each type of cancer. As shown in the table, several lncRNAs and pseudogenes are included in the top miRNA–RNA pairs, which corroborates the assertion that miRNAs play an important role in cancer through the interaction with lncRNAs and pseudogenes as well as with mRNAs [20,21]. If the higher |ΔPCC| of a miRNA–RNA pair is associated with a longer survival time, its hazard ratio (HR) < 1. In contrast, HR of a miRNA–RNA pair > 1 if the higher |ΔPCC| of the pair is associated with a shorter survival time.

**Figure 2:**
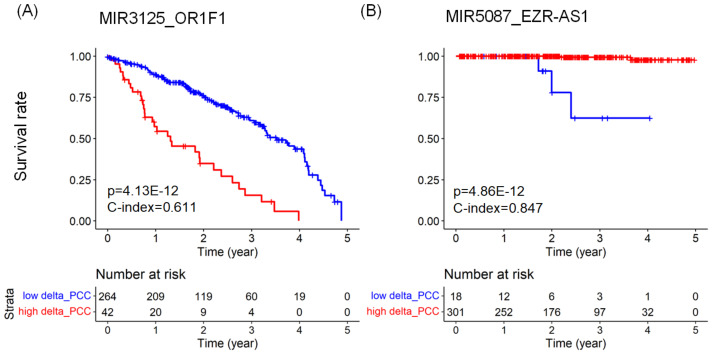
Kaplan–Meier plots comparing the survival rates of two groups of cancer patients with respect to a miRNA–RNA pair. (**A**) The survival rates of two groups of LUAD patients with respect to MIR3125_OR1F1. The larger |ΔPCC| values of the MIR3125_OR1F1 pair are associated with the shorter survival rates of LUAD patients. (**B**) The survival rates of two groups of PRAD patients with respect to MIR5087_EZR-AS1. The larger |ΔPCC| values of the MIR5087_EZR-AS1 pair are associated with the longer survival rates of PRAD patients. The risk tables below the Kaplan–Meier plots show the numbers at risk of each group over five years.

Figure 2 shows Kaplan–Meier plots and risk tables for the top miRNA–RNA pairs in LUAD and PRAD. In the Kaplan–Meier plots, the red line represents a group of patients with higher |ΔPCCs| than the threshold value. In contrast, the blue line represents a group of patients with lower |ΔPCCs| than the threshold value. The risk table below the Kaplan–Meier plot shows the number of patients at risk at a specific time point.

We examined how many of the miRNA–RNA pairs with an adjusted *p*-value < 0.01 in the log–rank test (available in Appendix B) are supported by existing experimental results or previously predicted using computational methods. For this comparison, we extracted miRNA–RNA interactions in humans from the RNAInter database [22], which provides a comprehensive RNA interactome resource, including miRNA–target RNA interactions. Among the 2322 miRNA–RNA pairs of Appendix B, 53 pairs were found as experimentally validated miRNA–RNA pairs in RNAInter, and 90 pairs were found as previously predicted pairs in RNAInter. Except the 143 pairs (53 experimentally validated pairs and 90 predicted pairs), most miRNA–RNA pairs found in our study were not found in RNAInter. This implies that our approach can be useful in finding previously unknown miRNA–RNA interactions.

### 2.4. Subnetworks for the Cancer Prognosis

With the miRNA–RNA pairs, we constructed star-shaped networks centered on common miRNAs, and selected the networks with C-index > 0.6, and adjusted *p*-value < 0.01. Two networks were found in BLCA, 14 in BRCA, 10 in COAD, 34 in ESCA, 1 in HNSC, 39 in LUAD, 1 in LUSC, 19 in PRAD, 2 in STAD, and 31 in THCA. The networks were named after their center nodes (e.g., network_MIR645 in LUAD, network_MIR4666A in PRAD).

Table 6 shows the top prognostic network biomarkers with the lowest *p*-value in the log–rank test. MIR145, which is present in the potential prognostic network biomarker of BLCA, is known as a potential biomarker of cancer migration and invasion [23]. MIR645 in the potential prognostic network biomarker of LUAD, is known to promote the proliferation of non-small cell lung cancer cells by targeting TP53I11 gene [24]. MIR760 in the prognostic network biomarker of STAD has been reported to function as a tumor suppressor and inhibit cell migration in gastric cancer in several studies [25,26]. MIR138 found for THCA is known to act as a tumor suppressor by targeting several genes that are related to the proliferation and invasion of cancer cells [27].

**Figure 3:**
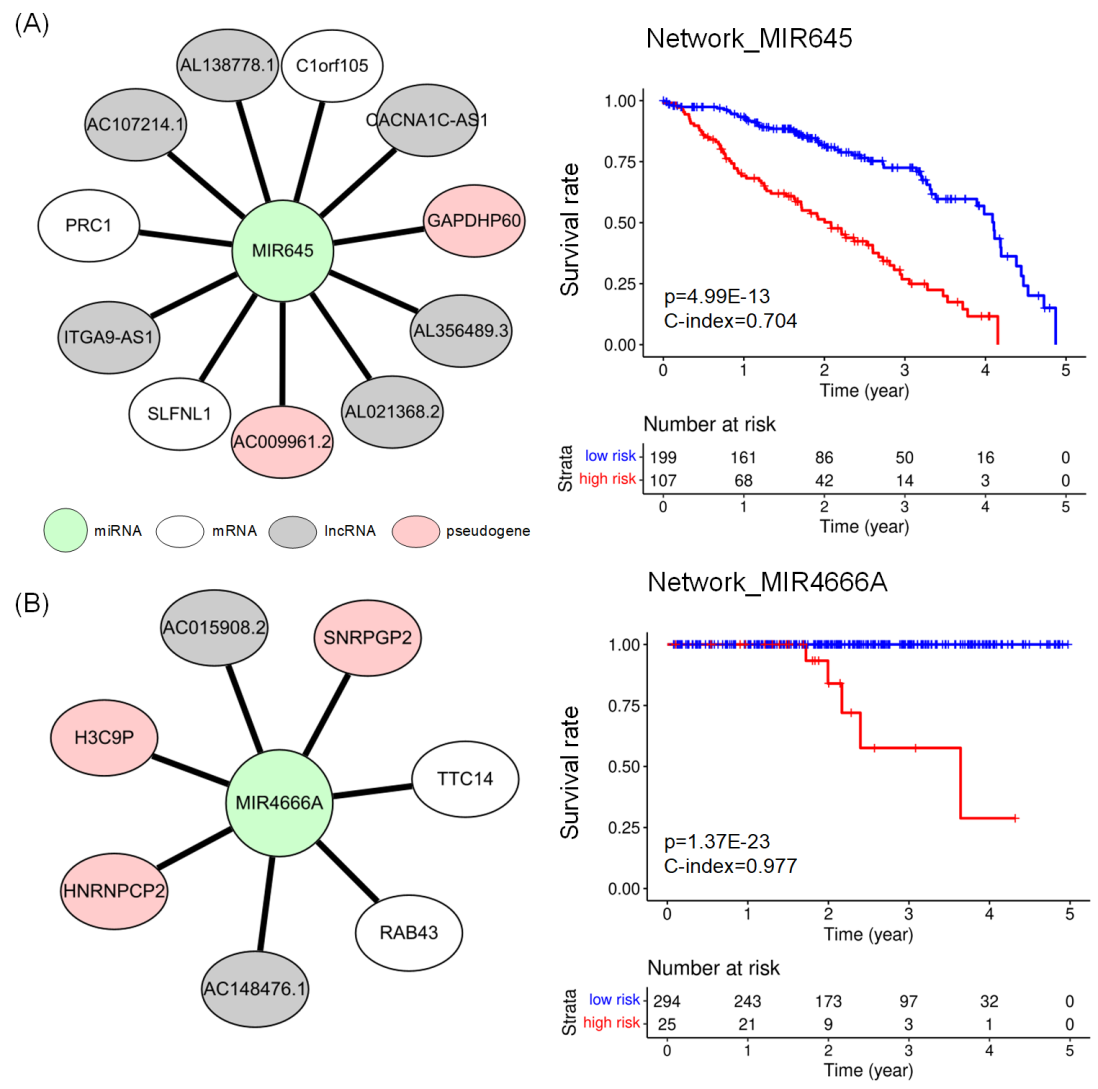
(**A**) Network_MIR645 for prognosis of LUAD, which consists of 3 mRNAs (white ellipse), 6 lncRNAs (grey ellipse), and 2 pseudogenes (pink ellipse) connected to 1 miRNA (shown as green circle). (**B**) Network_MIR4666A for prognosis of PRAD. It consists of 2 mRNAs, 2 lncRNAs, and 3 pseudogenes connected with miRNA. The Kaplan–Meier plots compare the survival rates of two groups of risk scores, which were defined using Equation (4).

Figure 3 shows the network biomarkers for LUAD and PRAD and the results of a survival analysis with the network biomarkers. The network_MIR645 (Figure 3A) consisting of 12 nodes (1 miRNA, 3 mRNAs, 6 lncRNAs, and 2 pseudogenes) revealed the lowest *p*-value in the log–rank test in LUAD. The network_MIR4666A (Figure 3B) includes 8 nodes (1 miRNA, 2 mRNAs, 2 lncRNAs, and 3 pseudogenes) showed the lowest *p*-value in the log–rank test and the highest C-index in PRAD. Detailed results of survival analysis with potential prognostic networks are available in Appendix C.

**Figure 4:**
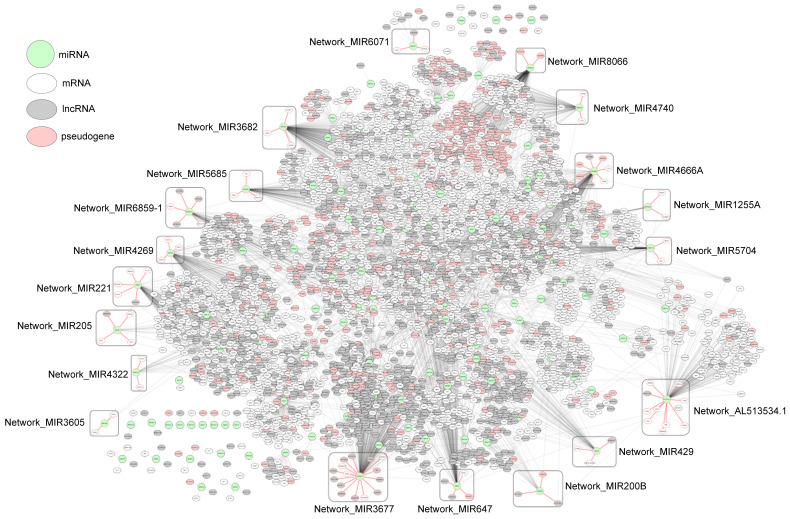
Network of miRNA–RNA correlations in PRAD, which consists of 5036 edges between 4121 nodes (125 miRNAs, 2330 mRNAs, 1169 lncRNAs, and 479 pseudogenes). Subnetworks enclosed with rounded boxes are potential prognostic network biomarkers found in our study.

As an example of miRNA–RNA correlation networks, Figure 4 shows a network composed of the miRNA–RNA pairs left after the Wilcoxon test in PRAD. The network consists of 5036 edges between 4121 nodes (125 miRNAs, 2330 mRNAs, 1169 lncRNAs, and 479 pseudogenes), and each edge represents ΔPCC of a miRNA–RNA pair. In the network, 19 potential prognostic network biomarkers of PRAD are enclosed with rounded boxes.

### 2.5. Comparing Potential Prognostic Biomarkers to Other Methods

We compared the prognostic power of the networks with that of miRNA–RNA pairs and individual genes in the networks in terms of the *p*-value of the log–rank test and C-index. Survival analysis with individual genes was based on the expression of the genes. For a fair comparison, we carried out the log–rank test for individual genes with an optimal threshold determined by the cutp function, as in the networks and miRNA–RNA pairs. We then selected individual genes with an adjusted *p*-value of the log–rank test < 0.01.

**Figure 5:**
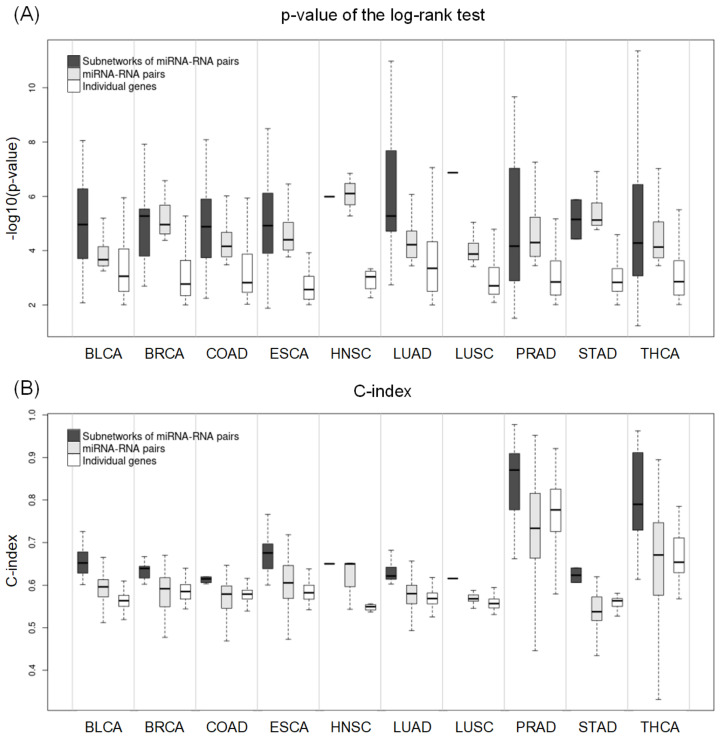
(**A**) Distribution of the *p*-values derived from the log–rank test with respect to subnetworks, miRNA–RNA pairs, and individual genes. (**B**) Distribution of the C-index values with respect to subnetworks, miRNA–RNA pairs, and individual genes.

Figure 5 shows the distribution of *p*-values of the log–rank test and C-index values of networks of miRNA–RNA pairs, miRNA–RNA pairs, and individual genes. In most cancer types, the best *p*-values, and C-indices were observed in network biomarkers, followed by miRNA–RNA pairs. In particular, the superiority of network biomarkers was prominent in C-index.

For more comparison, we selected the best network biomarker, miRNA–RNA pair, and gene and compared them in terms of the *p*-value of the log–rank test and C-index (Table 7). In all cancer types except BRCA and HNSC, networks of miRNA–RNA pairs were better than miRNA–RNA pairs and individual genes both in *p*-values and C-index. In BRCA and HNSC, miRNA–RNA pairs were the best, followed by networks of miRNA–RNA pairs. Overall, network biomarkers showed stronger prognostic power than miRNA–RNA pairs or individual genes in most cancer types.

We further compared the predictive power of our network biomarkers with the prognostic genes in the Human Protein Atlas (HPA) [28], which provides the results of the log–rank test with TCGA data sets, the same data sets used in our study. Since HPA does not provide C-index values of prognostic genes, we computed them with TCGA data sets. Table 8 compares 10 network biomarkers with the prognostic genes of HPA in terms of the *p*-values of the log–rank test and C-indices. Both the network biomarkers and the prognostic genes of HPA are the ones with the highest C-index in each type of cancer. A comparison of prognostic markers in ESCA was not made because HPA does not provide prognostic genes in ESCA. As shown in the table, the network biomarkers found in our study were better than prognostic genes of HPA not only in *p*-values but also in C-indices, with the exception of the *p*-value in BRCA.

## 3. Materials and Methods

### 3.1. Data Collection and Preparation

In the Cancer Genome Atlas (TCGA), we selected the data sets which have at least 50 tumor samples with lymph node metastasis (LNM) and 10 normal samples. Distant metastasis (DM) was not included in the selection criteria due to the small number of samples with distant metastasis. Among the 33 cancer data sets of TCGA, 10 cancer data sets satisfied the selection criteria: urothelial bladder carcinoma (BLCA), breast invasive carcinoma (BRCA), colon adenocarcinoma (COAD), esophageal carcinoma (ESCA), head-neck squamous cell carcinoma (HNSC), lung adenocarcinoma (LUAD), lung squamous cell carcinoma (LUSC), prostate adenocarcinoma (PRAD), stomach adenocarcinoma (STAD), and thyroid carcinoma (THCA).

The tumor samples in the selected cancer data sets were classified into four types based on the Tumor, Node, Metastasis (TNM) stage index in the clinical supplement data of TCGA.

- Samples with no metastasis (nonM): T stage of 1–4, N stage of 0, and M stage of 0
- Samples with lymph node metastasis only (LNM_only): T stage of 1–4, N stage of 1–3, and M stage of 0
- Samples with distant metastasis only (DM_only): T stage of 1–4, N stage of 0, and M stage of 1
- Samples with both lymph node metastasis and distant metastasis (LNM&DM): T stage of 1–4, N stage of 1–3, and M stage of 1

We obtained RNA-seq gene expression data from the Genomic Data Commons (GDC) data portal [29]. After filtering out the genes with the average raw read counts <1, a total of 42,692 genes were left. Using the annotation file obtained from the Ensembl project [30], we classified the remaining genes into 4 biotypes: miRNAs, lncRNAs, mRNAs, and pseudogenes. There were 42,692 genes used (477 miRNAs, 13,731 lncRNAs, 18,937 mRNAs, and 9547 pseudogenes) across 10 types of cancer. We then normalized raw read counts of the genes into counts per million (CPM) values using the trimmed mean of M values (TMM) method [31] in the R package edgeR [32].

### 3.2. Deriving miRNA–RNA Interactions

Our approach to predicting metastasis and prognosis is based on correlations of miRNAs and their target RNAs, which include mRNAs, lncRNAs, and pseudogenes. The correlations of miRNAs and their target RNAs were computed separately in each type of cancer. For every pair of miRNA and their target RNA in *n* normal samples, we computed the Pearson correlation coefficient (PCC) using Equation (1). In the equation, $X_{i}$ is the CPM value of miRNA X in sample *i*, and $\bar{X}$ is the mean CPM value of miRNA X in *n* samples. Likewise, $Y_{i}$ represents the CPM value of RNA Y in sample *i*, and $\bar{Y}$ is the mean CPM value of RNA Y in *n* samples.

(1) $$PCC\left(X,Y\right)=\frac{\sum_{i=1}^{n}\left(X_{i}-\bar{X}\right)\left(Y_{i}-\bar{Y}\right)}{\sqrt{\sum_{i=1}^{n}{\left(x_{i}-\bar{X}\right)}^{2}\sum_{i=1}^{n}{\left(Y_{i}-\bar{Y}\right)}^{2}}}$$

Our method for predicting metastasis is composed of two prediction models: one model for predicting lymph node metastasis ($M_{\mathrm{LNM}}$) and another model for predicting distant metastasis ($M_{\mathrm{DM}}$). In $M_{\mathrm{LNM}}$, LNM_only ∪ LNM&DM samples are positive, and nonM samples are negative. In $M_{\mathrm{DM}}$, DM_only ∪ LNM&DM samples are positive, and nonM ∪ LNM_only samples are negative.

In each of the positive and negative sets, miRNA–RNA pairs with |PCC(X,Y)| < 0.4 were removed because their correlations are not strong enough to be used in predicting metastasis. Those miRNA–RNA pairs common to the positive and negative data sets were also removed. After adding a single tumor sample to the *n* normal samples, we recomputed $PCC_{n+1}\left(X,Y\right)$ and obtained ΔPCC(X,Y) by subtracting $PCC_{n}$(X,Y) from $PCC_{n+1}\left(X,Y\right)$. ΔPCC(X,Y) reflects the difference in miRNA–RNA correlations between the *n* normal samples and the single tumor sample.

(2) $$\Delta PCC\left(X,Y\right)=PCC_{n+1}\left(X,Y\right)-PCC_{n}\left(X,Y\right)$$

Using the ΔPCC values, we performed the Wilcoxon test [33] between positive and negative data sets, and selected the miRNA–RNA pairs with the *p*-value < 0.01 in the Wilcoxon test. The miRNA–RNA pairs left after the Wilcoxon test represent those miRNA–RNA pairs with significantly different correlations (i.e., ΔPCC of a miRNA–RNA pair) in cancer patients.

### 3.3. Construction of Models for Predicting Metastasis

Gene expressions observed in lymph node metastasis are often different from those in distant metastasis, so predicting both types of metastasis with a single model is difficult [34]. Thus, our method is composed of two prediction models: one model for predicting lymph node metastasis ($M_{\mathrm{LNM}}$) and another model for predicting distant metastasis ($M_{\mathrm{DM}}$).

Both models use ΔPCC values of miRNA–RNA pairs as features, but the dimension of feature vectors was reduced by performing the principal component analysis (PCA). The models are ensemble learners with two base learners: support vector machine (SVM) with the radial basis function (RBF) as a kernel and logistic regression (LR). Using LR as a secondary learner, we combined the base learners by stacking to improve predictive accuracy [35,36].

The data sets were randomly partitioned into training and test data sets with a ratio of 7:3. The training data set and the test data set are disjoint. The test data set was used in independent testing. Due to the randomness of the data partition and the small and imbalanced data sets, the whole process of the data partition, training, and testing was repeated 10 times when evaluating the models. The hyperparameters of SVMs and LRs were optimized with a grid search with 5-fold cross-validation of training data sets.

**Figure 6:**
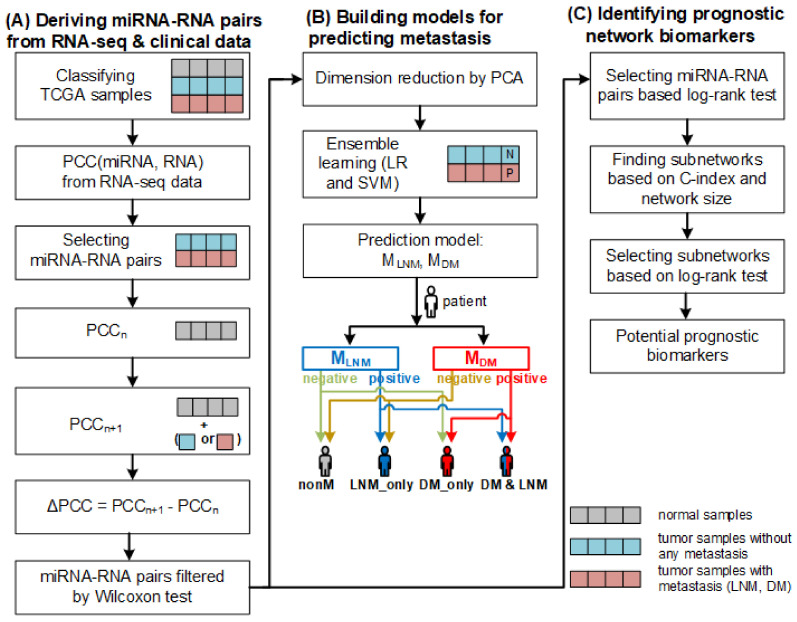
The overall framework of our method. (**A**) Deriving miRNA–RNA pairs based on ΔPCC from RNA–seq and clinical data. (**B**) Constructing prediction two models for predicting metastasis (LNM and DM) based on differential correlations between miRNAs and their target RNAs and predicting metastasis using the models. (**C**) Identifying prognostic network biomarkers from miRNA–RNA pairs.

The models take a patient sample as input. If both models classify the sample as negative, no metastasis is predicted for the patient. If the sample is classified as positive by $M_{\mathrm{LNM}}$ but negative by $M_{\mathrm{DM}}$, only lymph node metastasis is predicted for the patient. Similarly, if the sample is classified as negative by $M_{\mathrm{LNM}}$ but positive by $M_{\mathrm{DM}}$, only distant metastasis is predicted for the patient. If both models classify the sample as positive, both lymph node metastasis and distant metastasis are predicted for the patient (refer to Figure 6).

The overall workflow of constructing the prediction models and running them is illustrated in Figure 6. Constructing the models involves data collection, classifying samples, deriving miRNA–RNA pairs, computing differential correlations of miRNA–RNA pairs, and training the models.

### 3.4. Finding Biomarkers for Predicting Prognosis

We used the miRNA–RNA pairs derived for predicting metastasis in finding prognostic biomarkers. The workflow of finding prognostic biomarkers is illustrated in Figure 6. We derived two types of prognostic biomarkers: miRNA–RNA pair and subnetwork centered at a common miRNA of miRNA–RNA pairs. We carried out the univariate Cox regression analysis [37] with |ΔPCC| values of miRNA–RNA pairs and computed the concordance index (C-index) of every miRNA–RNA pair. The C-index for every pair in patient samples *i* and *j* is defined using Equation (3), where $T_{i}$ is an observed survival time of *i* and $\eta_{i}$ is a predicted score of *i*. $\eta_{i}$ could be predicted survival times, or hazards, etc. In this study, partial hazard values predicted with the Cox regression model were used as $\eta_{i}$ [38].

(3) $$\begin{array}{cc} C-\mathrm{index} & =\frac{\sum_{i\neq j}^{}\delta \left(T_{i}>T_{j}\right)\cdot \delta \left(\eta_{i}<\eta_{j}\right)\cdot d_{j}}{\sum_{i\neq j}^{}\delta \left(T_{i}>T_{j}\right)\cdot d_{j}} \end{array}$$

where $d_{j}$ = 1 if *j* is uncensored, and 0 otherwise. $\delta (T_{i}>T_{j})$ = 1 if $T_{i}>T_{j}$, and 0 otherwise. The C-index ranges between 0 and 1, 1 being the best value. We also performed the log–rank test for each miRNA–RNA pair. When dividing patient samples into two groups (high |ΔPCC| group and low |ΔPCC| group), we used the cutp function in the R package survMisc [39]. The cutp function determines an optimal cut point for a continuous variable based on the statistical results of the Cox regression analysis. We adjusted the *p*-values of the log–rank test using the Benjamini–Hochberg procedure [40], and selected the miRNA–RNA pairs with an adjusted *p*-value < 0.01 as potential prognostic miRNA–RNA pairs.

The miRNA–RNA pairs with an adjusted *p*-value < 0.01 were sorted in increasing order of *p*-values. Starting with the miRNA–RNA pair with the smallest *p*-value, we combined up to 15 miRNA–RNA pairs with common miRNAs. The combined miRNA–RNA pairs form star-shaped networks centered at common miRNAs.

For every patient sample *i*, we computed the risk score of the star-shaped networks using Equation (4). In Equation (4), *j* denotes a miRNA–RNA pair in a network. ${\left|\Delta \mathrm{PCC}\right|}_{j}^{i}$ represents the |ΔPCC| values of miRNA–RNA pair *j* in sample *i*. $\beta_{j}$ is the regression coefficient from the Cox regression analysis of miRNA–RNA pair *j*.

(4) $$\mathrm{Risk}\;\mathrm{score}\left(i\right)=\sum_{j}^{}{\left|\Delta \mathrm{PCC}\right|}_{j}^{i}\beta_{j}$$

The risk score was used in classifying patient samples into two groups, the high-risk group and the low-risk group. Again, the cutp function was used to determine an optimal threshold for classification. Finally, the networks with a C-index > 0.6 and adjusted *p*-value < 0.01 were selected as potential prognostic biomarkers.

## 4. Conclusions

So far, many computational methods for predicting prognosis in cancer have focused on survival rates without considering metastasis. There are a few methods developed for predicting lymph node metastasis, but few attempts have been made to predict distant metastasis mainly due to the difficulty of the problem and the small number of publicly available samples with distant metastasis. We developed a new method for predicting both lymph node metastasis and distant metastasis using differential correlations of miRNAs and their target RNAs in cancer, which were derived from a large amount of RNA-seq data and clinical data. Testing our method on several types of cancer demonstrated that differential correlations of miRNAs and their target RNAs are much more powerful than gene expressions in predicting distant metastasis as well as lymph node metastasis. With the differential correlations of miRNAs and their target RNAs, we found network biomarkers for predicting the prognosis of cancer patients. The network biomarkers derived from metastasis analysis were more predictive of survival rates than correlations of individual miRNA–RNA pairs or gene expressions of individual genes. The results of our study showed that network biomarkers based on correlations of genes could be more powerful than typical molecular biomarkers of individual genes in predicting prognosis as well as metastasis. The method developed in this study, and its results will be useful in selecting treatment options for cancer patients and a target of anti-cancer drug discovery.

## Figures and Tables

**Table 1:** The number of tumor samples of each type and normal samples in 10 cancer data sets.

Cancer	#nonM samples	#LNM_only samples	#DM_only samples	#LNM&DM samples	#normal samples	#total samples
BLCA	118	44	0	7	19	188
BRCA	450	449	1	18	113	1031
COAD	228	105	9	55	41	438
ESCA	56	64	2	6	11	139
HNSC	81	98	0	1	44	224
LUAD	219	124	11	11	59	424
LUSC	258	149	3	3	49	462
PRAD	316	75	1	1	52	445
STAD	103	210	2	23	32	370
THCA	145	127	3	4	59	338

**Table 2:** The number of miRNAs, lncRNAs, mRNAs, and pseudogenes in ten types of cancer.

Cancer	#miRNAs	#lncRNAs	#mRNAs	#pseudogenes
BLCA	143	9612	18,038	4994
BRCA	150	10,070	18,035	5380
COAD	144	8477	17,515	5102
ESCA	418	12,588	18,658	8713
HNSC	88	8563	17,912	4493
LUAD	182	10,291	18,037	5845
LUSC	147	10,206	18,152	5507
PRAD	126	8764	17,731	4686
STAD	345	12,472	18,657	8700
THCA	140	8256	17,487	4610

**Table 3:** The number of features after each filtering process.

Cancer	#miRNA–RNA Pairs after PCC Filtering	#miRNA–RNA Pairs after Wilcoxon Test	#PCs after PCA
LNM			
BLCA	169,439	9501	45
BRCA	170,673	3619	166
COAD	312,968	3970	162
ESCA	706,722	27,312	65
HNSC	82,959	2281	58
LUAD	320,323	13,891	137
LUSC	43,340	1296	83
PRAD	78,722	5036	150
STAD	230,038	8136	120
THCA	124,722	12,738	102
DM			
BRCA	572,862	19,634	134
COAD	273,660	4968	112
LUAD	863,846	20,632	55
STAD	1,222,396	43,240	58

**Table 4:** Comparison of three types of features in predicting lymph node metastasis (LNM) and distant metastasis (DM) with respect of AUC in independent testing. All the values are the average of 10 runs. Bold values indicate best values. -: no result.

Cancer	LNM			DM		
	ΔPCC ^1^	Exp ^2^	Exp191 ^3^	ΔPCC ^1^	Exp ^2^	Exp191 ^3^
BLCA	**0.938**	0.668	0.541	-	-	-
BRCA	**0.732**	0.626	0.550	**0.907**	0.605	0.500
COAD	**0.936**	0.713	0.637	**0.889**	0.580	0.512
ESCA	**0.961**	0.670	0.501	-	-	-
HNSC	**0.924**	0.727	0.520	-	-	-
LUAD	**0.787**	0.636	0.557	**0.733**	0.613	0.500
LUSC	**0.840**	0.598	0.498	-	-	-
PRAD	**0.815**	0.655	0.534	-	-	-
STAD	**0.897**	0.596	0.507	**0.853**	0.661	0.498
THCA	**0.802**	0.675	0.638	-	-	-

^1^ ΔPCC of miRNA–RNA pairs, ^2^ Expressions of genes involved in miRNA–RNA pairs, ^3^ Expressions of 191 metastasis-predictive genes [19].

**Table 5:** The results of univariate Cox regression analysis with respect to miRNA–RNA pairs with the lowest *p*-value in the log–rank test. HR: hazard ratio.

Cancer	miRNA–RNA Pair	Type of RNA	HR	*p*-Value	C-Index
BLCA	MIR6793_CST4	mRNA	0.164	$1.53\times 10^{-10}$	0.639
BRCA	MIR186_AP1S1	mRNA	3.820	$6.48\times 10^{-9}$	0.642
COAD	MIR4538_SLAMF1	mRNA	3.294	$4.89\times 10^{-8}$	0.630
ESCA	MIR4755_CCDC18-AS1	lncRNA	5.298	$5.63\times 10^{-9}$	0.681
HNSC	MIR4537_EMC3-AS1	pseudogene	0.256	$1.42\times 10^{-7}$	0.651
LUAD	MIR3125_OR1F1	mRNA	3.868	$4.13\times 10^{-12}$	0.611
LUSC	MIR6071_SFTA3	lncRNA	0.408	$3.34\times 10^{-6}$	0.579
PRAD	MIR5087_EZR-AS1	lncRNA	0.022	$4.86\times 10^{-12}$	0.847
STAD	MIR6757_AC104619.3	pseudogene	5.724	$4.23\times 10^{-9}$	0.537
THCA	MIR4664_AL353138.1	lncRNA	0.014	$6.19\times 10^{-13}$	0.863

**Table 6:** The top subnetworks with the lowest *p*-value of the log-rank test for each type of cancer.

Cancer	Network	#edges	HR	*p*-Value	C-Index
BLCA	network_MIR145	15	7.476	$3.26\times 10^{-11}$	0.710
BRCA	network_MIR378J	3	3.357	$1.19\times 10^{-8}$	0.638
COAD	network_MIR4538	15	3.491	$8.13\times 10^{-9}$	0.689
ESCA	network_MIR4644	15	6.312	$3.21\times 10^{-9}$	0.788
HNSC	network_MIR8058	2	4.146	$1.02\times 10^{-6}$	0.650
LUAD	network_MIR645	11	3.628	$4.99\times 10^{-13}$	0.704
LUSC	network_MIR6071	15	2.400	$1.34\times 10^{-7}$	0.615
PRAD	network_MIR4666A	7	$2.775\times 10^{10}$	$1.37\times 10^{-23}$	0.977
STAD	network_MIR760	5	2.325	$1.33\times 10^{-6}$	0.640
THCA	network_MIR138-1	2	46.806	$1.72\times 10^{-15}$	0.789

**Table 7:** The number of features and the best *p*-value and C-index for each cancer type in subnetworks, miRNA–RNA pairs, and individual genes. Bold values indicate best values.

Cancer	Type of Feature	Number of Features	*p*-Value	C-Index
BLCA	networks	32	$3.26\times 10^{-11}$	**0.7264**
	miRNA–RNA pairs	514	$1.53\times 10^{-10}$	0.6656
	individual genes	297	$2.56\times 10^{-7}$	0.6100
BRCA	networks	14	$1.19\times 10^{-8}$	0.6673
	miRNA–RNA pairs	93	$6.48\times 10^{-9}$	**0.6701**
	individual genes	52	$6.74\times 10^{-7}$	0.6396
COAD	networks	10	$8.13\times 10^{-9}$	**0.6895**
	miRNA–RNA pairs	190	$4.89\times 10^{-8}$	0.6470
	individual genes	100	$2.96\times 10^{-8}$	0.6192
ESCA	networks	34	$3.21\times 10^{-9}$	**0.7888**
	miRNA–RNA pairs	311	$5.63\times 10^{-9}$	0.7185
	individual genes	98	$1.28\times 10^{-5}$	0.6384
HNSC	networks	1	$1.02\times 10^{-6}$	0.6502
	miRNA–RNA pairs	3	$1.42\times 10^{-7}$	**0.6516**
	individual genes	4	$0.04\times 10^{-2}$	0.5562
LUAD	networks	39	$4.99\times 10^{-13}$	**0.7154**
	miRNA–RNA pairs	632	$4.13\times 10^{-12}$	0.6565
	individual genes	342	$2.44\times 10^{-10}$	0.6242
LUSC	networks	1	$1.34\times 10^{-7}$	**0.6157**
	miRNA–RNA pairs	53	$3.34\times 10^{-6}$	0.5875
	individual genes	42	$3.85\times 10^{-6}$	0.5943
PRAD	networks	19	$1.37\times 10^{-23}$	**0.9773**
	miRNA–RNA pairs	156	$4.86\times 10^{-12}$	0.9519
	individual genes	66	$1.06\times 10^{-13}$	0.9210
STAD	networks	2	$1.33\times 10^{-6}$	**0.6406**
	miRNA–RNA pairs	77	$4.23\times 10^{-9}$	0.6196
	individual genes	39	$3.28\times 10^{-6}$	0.6135
THCA	networks	31	$1.72\times 10^{-15}$	**0.9627**
	miRNA–RNA pairs	293	$6.19\times 10^{-13}$	0.8950
	individual genes	78	$1.76\times 10^{-11}$	0.7854

**Table 8:** Comparison of the proposed prognostic networks centered on miRNAs and the prognostic genes of HPA. Both prognostic networks found in our study and the prognostic genes of HPA are the ones with the highest C-index. Bold values indicate best values.

Cancer	Prognostic Networks Found in Our Study			Prognostic Genes in HPA		
	Center miRNA	*p*-Value	C-Index	Gene	*p*-Value	C-Index
BLCA	MIR4539	$6.89\times 10^{-7}$	**0.7264**	GARS1	$3.04\times 10^{-5}$	0.6226
BRCA	MIR4489	$3.09\times 10^{-7}$	**0.6673**	PGK1	$7.05\times 10^{-8}$	0.6580
COAD	MIR4538	$8.13\times 10^{-9}$	**0.6895**	PRKAR2A	$4.27\times 10^{-5}$	0.6411
ESCA	MIR4644	$3.21\times 10^{-9}$	0.7888	-	-	-
HNSC	MIR8058	$1.02\times 10^{-6}$	**0.6502**	IGHV3-13	$6.67\times 10^{-6}$	0.6133
LUAD	MIR624	$1.05\times 10^{-11}$	**0.7154**	DKK1	$7.80\times 10^{-6}$	0.6480
LUSC	MIR6071	$1.34\times 10^{-7}$	**0.6157**	NT5E	$0.06\times 10^{-2}$	0.5987
PRAD	MIR466A	$1.37\times 10^{-23}$	**0.9773**	SESN1	$0.02\times 10^{-2}$	0.9029
STAD	MIR760	$1.33\times 10^{-6}$	**0.6406**	ZBTB7A	$0.02\times 10^{-2}$	0.6135
THCA	MIR4442	$1.77\times 10^{-8}$	**0.9627**	SNAI1	$8.82\times 10^{-7}$	0.8500

## Data Availability

The data in the Appendix A, Appendix B and Appendix C sections are available at http://bclab.inha.ac.kr/biomarker (accessed on 15 January 2023).

## Abbreviations

The following abbreviations are used in this manuscript:

TCGA	The cancer genome atlas
BLCA	Urothelial bladder carcinoma
BRCA	Breast invasive carcinoma
COAD	Colon adenocarcinoma
ESCA	Esophageal carcinoma
HNSC	Head-neck squamous cell carcinoma
LUAD	Lung adenocarcinoma
LUSC	Lung squamous cell carcinoma
PRAD	Prostate adenocarcinoma
STAD	Stomach adenocarcinoma
THCA	Thyroid carcinoma
TNM	Tumor, node, metastasis
GDC	Genomic data commons
CPM	Counts per million
TMM	Trimmed mean of M values
PCC	Pearson correlation coefficient
PCA	Principal component analysis
PC	Principal component
SVM	Support vector machine
RBF	Radial basis function
LR	Logistic regression
HR	Hazard ratio
AUC	Area under the curve
HPA	Human protein atlas

## Appendix A

Results of predicting metastasis in 10 types of cancer.

## Appendix B

miRNA–RNA pairs with an adjusted *p*-value less than 0.01 in the log–rank test.

## Appendix C

Potential prognostic networks in 10 types of cancer.
